# Kiesselbach’s area nasal septal gouty tophus with an integrated “3 + 2” diagnostic algorithm: a case report

**DOI:** 10.1186/s13256-025-05691-4

**Published:** 2025-11-28

**Authors:** Weikun Chen, Wanling Chen, Duanyue Guo, Ziyue Wang, Ziwen Shi, Junwei Zhong

**Affiliations:** 1 Department of Otolaryngology-Head and Neck Surgery, Shenshan Medical Center, Sun Yat-sen Memorial Hospital, Sun Yat-sen University, Shanwei, 516621 Guangdong People’s Republic of China; 2 Department of Radiology, Shenshan Medical Center, Sun Yat-sen Memorial Hospital, Sun Yat-sen University, Shanwei, 516621 Guangdong People’s Republic of China

**Keywords:** Gouty tophus, Kiesselbach’s area, Dual-energy computed tomography, Nasal mass, Urate-lowering therapy, Diagnostic algorithm, Case report

## Abstract

**Background:**

Gout, characterized by monosodium urate crystal deposition, rarely involves the nasal septum, with only a few reported cases. Such unusual presentations can mimic other conditions, necessitating prompt and accurate diagnosis. This report describes a rare case of gouty tophus in Kiesselbach’s area of the anterior nasal septum.

**Case presentation:**

A 59 year-old Han Chinese man with a 20/year history of gout, off urate-lowering therapy, presented with painless external nasal swelling. Imaging, including computed tomography (110–220 Hounsfield units calcified nodule) and dual-energy computed tomography (confirmed monosodium urate deposition), combined with histopathology, validated tophaceous gout. He underwent open surgical excision and received postoperative urate-lowering therapy, achieving no recurrence at 9 months with serum urate < 300 μmol/l.

**Conclusion:**

This case highlights the importance of considering gouty tophus in atypical nasal masses, especially in patients with gout history. Dual-energy computed tomography is a valuable diagnostic tool. We propose a practical “3 + 2” diagnostic algorithm to aid early recognition, although further validation in larger series is needed.

## Introduction

Gout is characterized by monosodium urate crystal deposition in joints and soft tissues. Nasal septal involvement is exceedingly rare, with only three cases reported to date [1–3]. The Kiesselbach’s plexus (Little’s area) on the anterior septum is the most vascularized region in the nasal cavity [4] and represents an unusual site for tophus formation. Because nasal tophi may mimic neoplastic or inflammatory lesions, prompt recognition is essential to guide appropriate therapy. We describe a case of nasal septal gouty tophus confirmed by dual-energy computed tomography (DECT) and histopathology, and we propose a “3 + 2” diagnostic algorithm to facilitate early diagnosis.

## Case presentation

A 59 year-old Han Chinese man presented to our clinic on 20 August 2024, with a 3-month history of progressive, painless swelling of the external nose. He had a significant medical history of gout spanning 20 years. He reported recurrent and often severe gout attacks, typically involving the bilateral great toes and ankles. Notably, he had no history of subcutaneous tophi in other common locations such as the elbows, helix of the ear, or olecranon bursa. He had unilaterally discontinued urate-lowering therapy (ULT) approximately 5 years prior due to perceived disease remission and poor adherence, leading to more frequent and intense acute gout flares in recent years. There was no significant family history of gout or hyperuricemia. His social history indicated occasional alcohol consumption and a diet rich in purines, factors commonly associated with gout exacerbations; however, he reported no other significant psychosocial issues. For the initial 3 months of the nasal swelling, he had not sought medical attention or attempted any self-treatment, attributing the painless nature of the mass to a benign cause before it became noticeably larger.

**Fig. 1:**
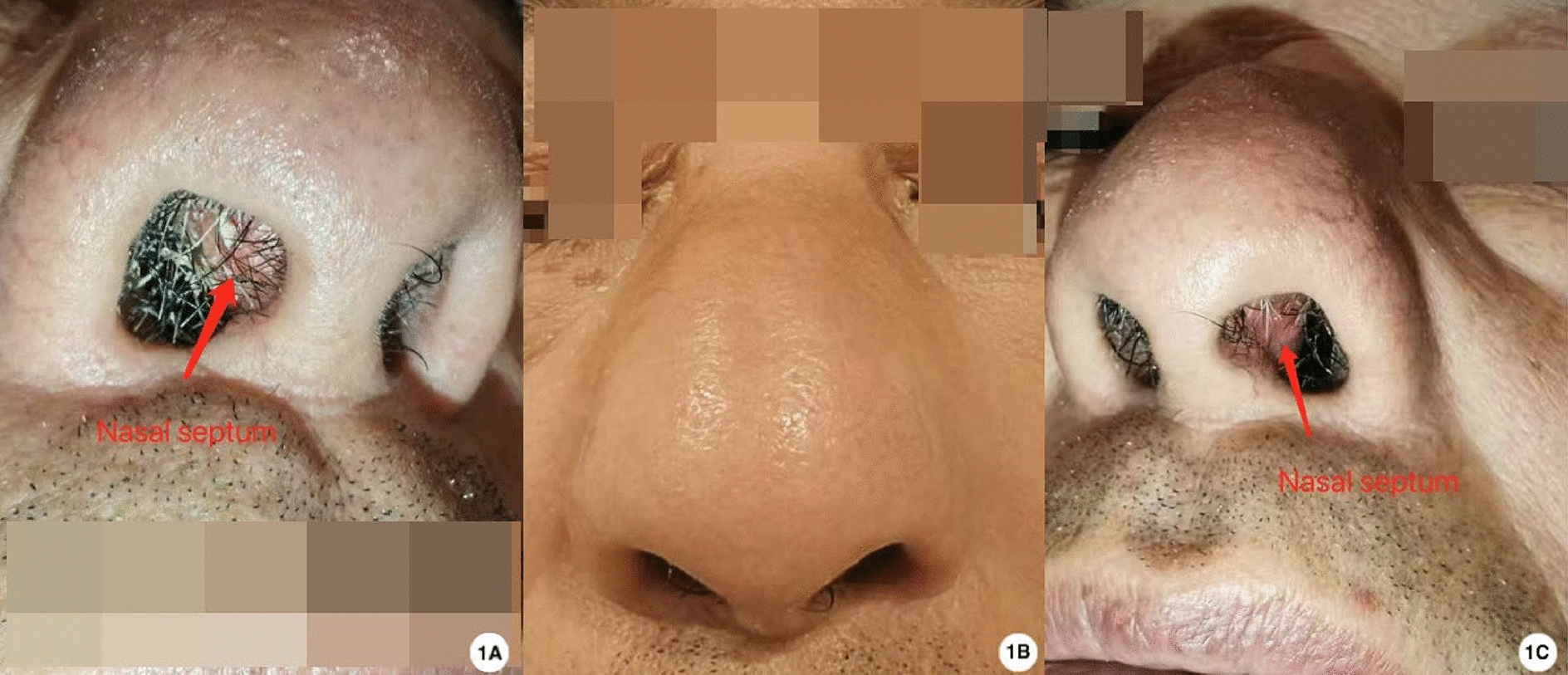
Clinical and endoscopic findings of the nasal septum. **B** External nasal tumefaction with **A**,**C** bilateral prominence of the nasal septum (arrow), which was palpably firm

**Fig. 2:**
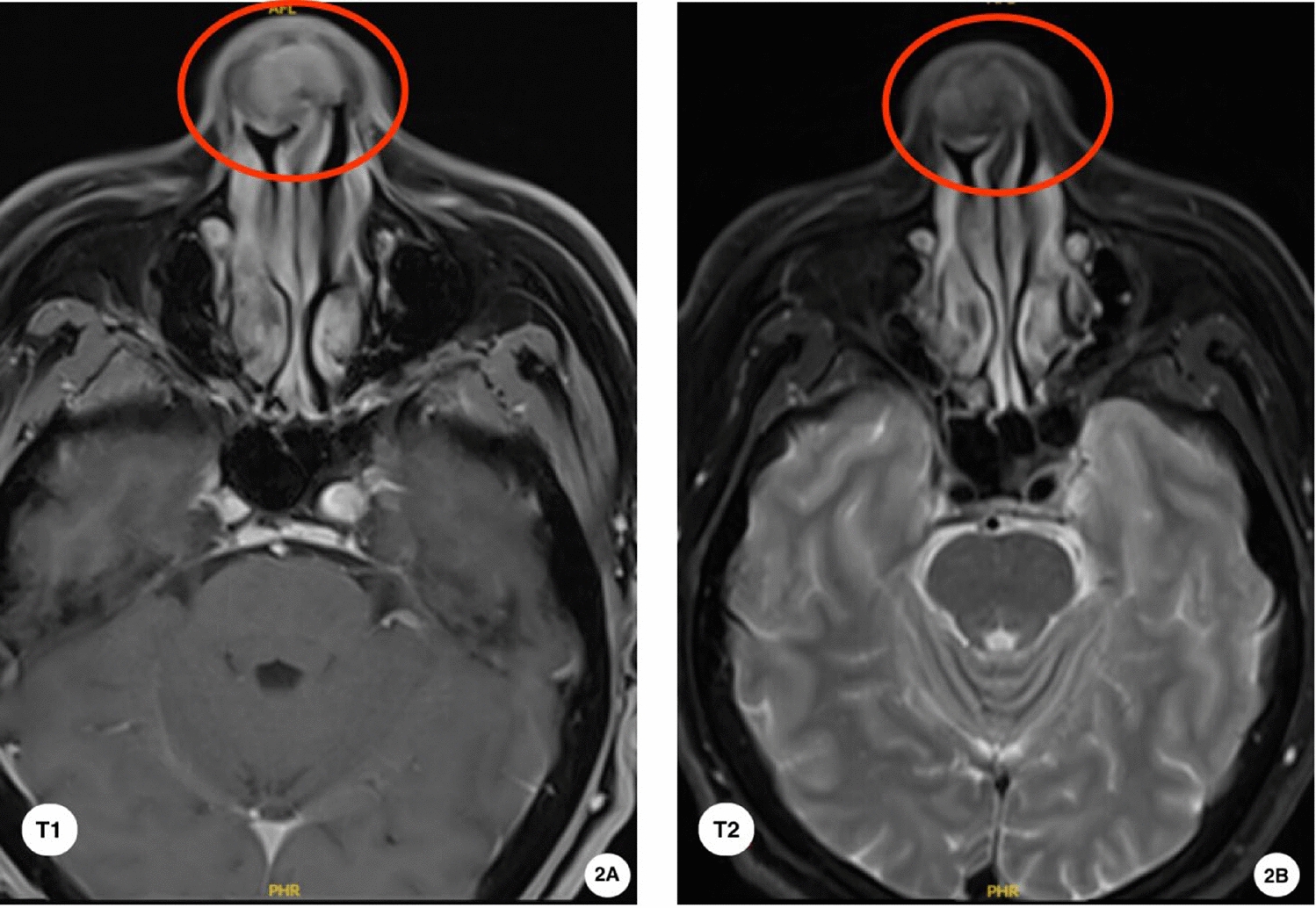
Nasal septum magnetic resonance imaging (axial view) findings. **A** T1 post-contrast image shows an enhancing hypointense nodule within the nasal septum. **B** T2-weighted image demonstrates a nasal septal nodule with heterogeneous signal intensity (mixed high and low signal). The red circles indicate the location of the lesion (gouty tophus) in the nasal septum

**Fig. 3:**
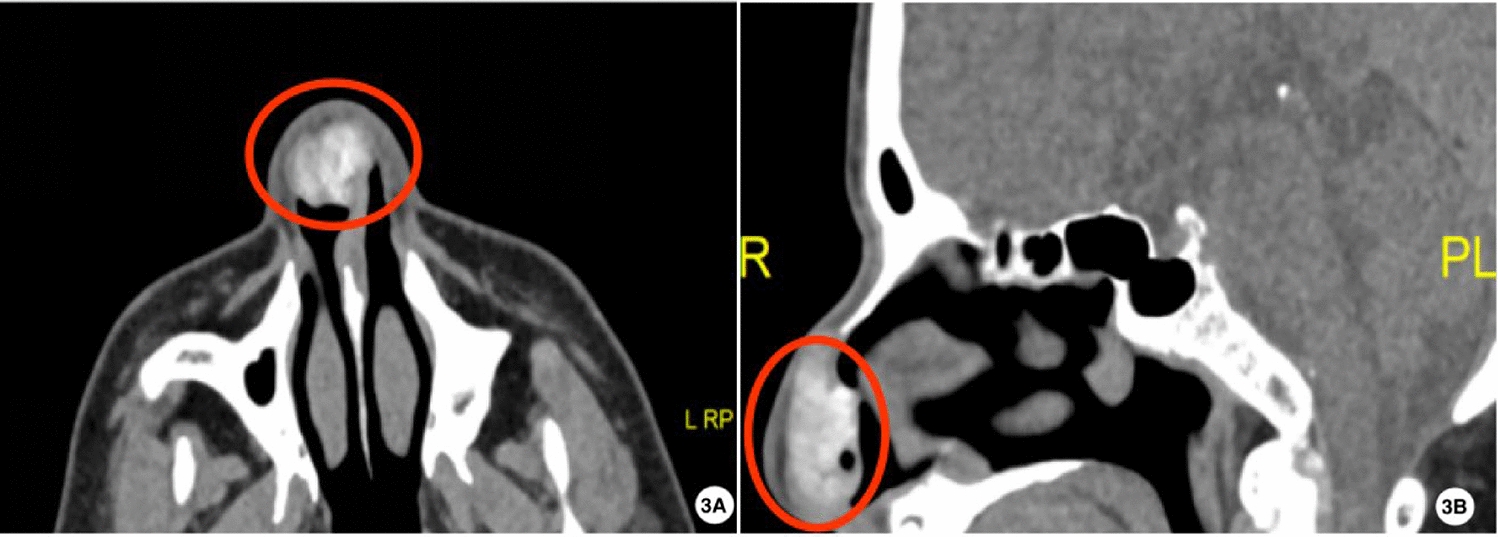
Axial computed tomography of nasal cavity (**A)** and sagittal computed tomography of nasal cavity (**B)**. Soft tissue nodule is observed in the anterior nasal septum, protruding into the right nasal vestibule. The nodule exhibits irregular calcification (circled). The lesion is well demarcated, measuring approximately 16 mm × 20 mm × 27 mm. The nodule’s attenuation values ranged from 110 to 220 Hounsfield units

**Fig. 4:**
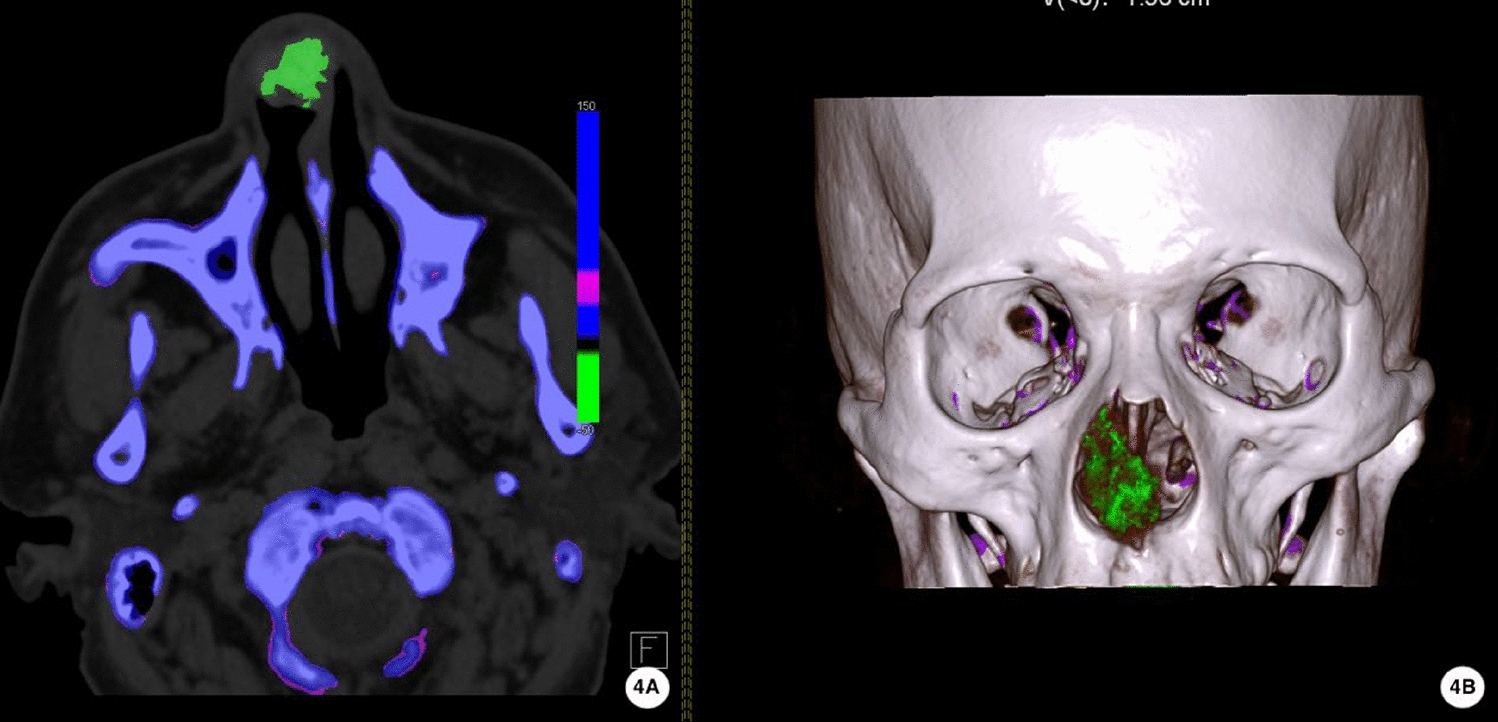
Dual-energy computed tomography (DECT) gout analysis (**A)** combined with bone three-dimensional reconstruction (**B)** demonstrating green color-coding as representing areas of urate (monosodium urate) crystal deposition

Upon initial examination on 20 August 2024, a firm, smooth 2.5 cm mass was observed over the nasal tip extending to the supratip area, with bilateral septal prominence but intact mucosa (Fig. 1). Serum uric acid was 430 μmol/l (reference 202–417 μmol/l). Magnetic resonance imaging (MRI) showed a well-circumscribed T1 hypointense, T2 hypointense nodule in the anterior septum with mild heterogeneous enhancement (Fig. 2). Noncontrast CT demonstrated a 16 × 20 × 27 mm soft-tissue mass with irregular calcifications and attenuation values of 110–220 Hounsfield units (HU) (Fig. 3). DECT color-coded voxels confirmed monosodium urate crystal deposition (Fig. 4).

**Fig. 5:**
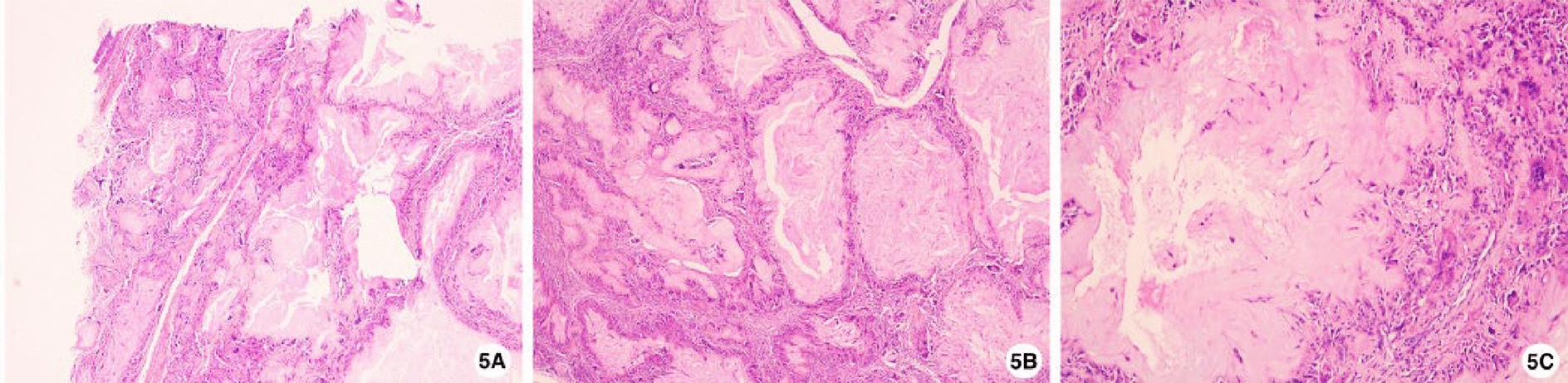
Pathological features confirming tophaceous gout: **A** Amorphous eosinophilic deposits with peripheral fibrosis and minimal chronic inflammation (hematoxylin and eosin, ×40). **B** Scant cellularity within these deposits, with spindle cell predominance (hematoxylin and eosin, ×40). **C** Characteristic cleft-like spaces in the deposits, surrounded by marked chronic inflammation and foreign body giant cells (urate tophi) (hematoxylin and eosin, ×100)

Following diagnostic confirmation, open surgical removal was performed via an infra-columellar modified butterfly incision. Careful subperichondrial dissection exposed a lobulated, firm mass adherent to septal cartilage, which was excised completely with minimal blood loss (< 10 ml). Histology showed amorphous eosinophilic deposits with peripheral fibrosis, foreign body giant cells, and characteristic cleft-like spaces, consistent with tophaceous gout (Fig. 5).

Postoperatively, the patient received colchicine 0.5 mg daily, febuxostat 40 mg daily, and potassium citrate granules 2.5 g twice daily. At 9-month follow-up, external nasal contour was normal, serum urate remained < 300 μmol/l, and CT showed no evidence of recurrence.

## Discussion

Nasal septal tophi are rare but should be considered in patients with gout presenting with nasal masses [1–3]. This report adds to the limited number of documented cases, highlighting an even rarer presentation within the highly vascularized Kiesselbach’s area, a region primarily known for epistaxis [5]. Imaging is crucial: CT may reveal calcified nodules with HU values of 110–220, while DECT specifically identifies urate crystals. Histopathology remains the gold standard.

Comparing our case with the few existing reports of nasal tophi, distinct features emerge [6]. While prior cases have sometimes involved bone erosion or presented as infected lesions [7], our patient presented with a painless, progressively enlarging soft tissue mass largely confined to the anterior nasal septum without overt bone destruction. This distinction underscores the varied presentations of nasal tophi and emphasizes the novelty of Kiesselbach’s area as a primary deposition site [4, 8], potentially influenced by its unique microenvironment.

**Fig. 6:**
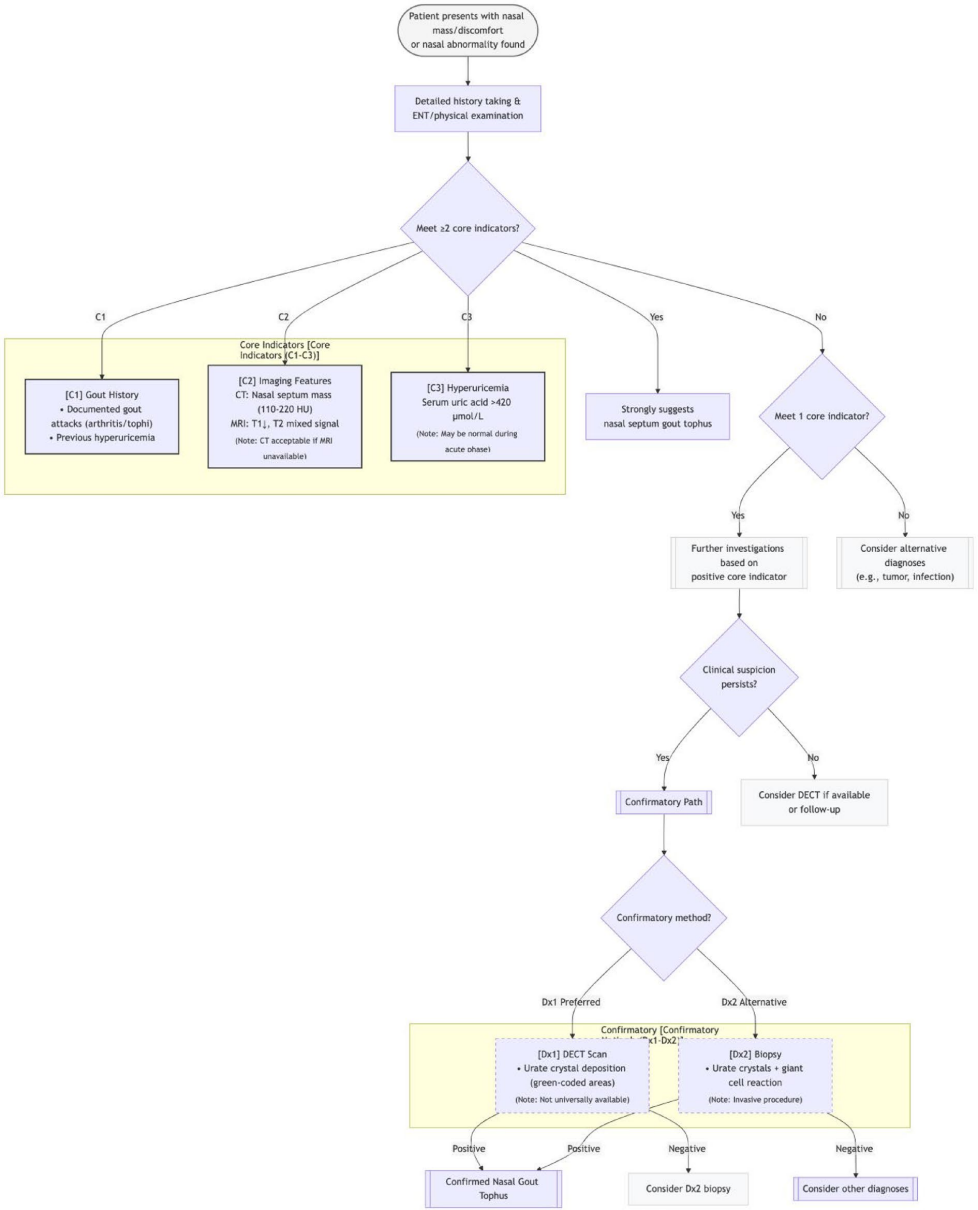
Proposed “3 + 2” diagnostic algorithm for nasal septal gouty tophus

We propose a “3 + 2” diagnostic algorithm (Fig. 6) to aid early recognition and guide management. The core indicators for screening include: history of gout; characteristic imaging features such as calcified nodules on CT (110–220 HU) or specific MRI signals; and presence of hyperuricemia. For definitive diagnosis, confirmatory indicators are required: dual-energy CT demonstration of urate deposition or histopathological confirmation. Patients meeting all core indicators should proceed with DECT or biopsy for definitive confirmation. This structured workflow seeks to balance rapid screening with diagnostic precision, thereby minimizing unnecessary invasive procedures. However, it is crucial to acknowledge that this proposed “3 + 2” diagnostic algorithm is based on a single case report, which inherently limits its generalizability[9]. Its potential weaknesses include limited applicability in patients without a clear history of gout or those who are normouricemic, where the “core indicators” might not be present [10]. Therefore, while it serves as a valuable preliminary screening and diagnostic aid, its definitive validation necessitates larger, multicenter studies across diverse patient populations to establish its efficacy and broader clinical utility.

Beyond diagnostic considerations, exploring the specific mechanisms underlying tophus formation in such an unusual anatomical site is also crucial. In this regard, the pathogenesis of tophus formation in Kiesselbach’s area may involve local hemodynamic factors. Computational fluid dynamics studies suggest that turbulent airflow and vortices in this region can reduce shear stress and potentially lower local pH, which may favor urate crystal precipitation [11, 12]. Confirmation of this hypothesis requires further experimental work. Further research into the specific microenvironmental factors, such as local temperature fluctuations and microtrauma within this highly vascularized area, could provide deeper insights into its susceptibility to urate deposition.

Given the diverse nature of nasal masses, it is imperative to consider a wide array of differential diagnoses when evaluating a lesion in this region. Specifically, differential diagnoses for nasal masses in this region are broad and include infections (for example, tuberculoma or invasive fungal sinusitis [13, 14]), benign neoplasms (for example, hemangioma [15], collagenous fibroma [16]), malignant tumors [17], and granulomatous diseases (for example, granulomatosis with polyangiitis or sarcoidosis [18, 19]). Other considerations for a nasal septal mass include nasal polyps, cysts, and idiopathic inflammatory pseudotumors. DECT plays a crucial role in distinguishing urate tophi from these other entities due to its specificity for urate crystals. In our case, the patient’s longstanding history of gout and hyperuricemia served as key clinical clues. DECT provided a noninvasive, highly specific method to identify monosodium urate deposition, directly differentiating it from other calcified or soft tissue lesions[20]. Subsequently, histopathological examination, revealing characteristic amorphous eosinophilic deposits with cleft-like spaces and foreign body giant cells, provided the definitive diagnosis, confirming the DECT findings and excluding other possibilities. Ultrasonic-guided fine-needle aspiration cytology (FNAC) could also be considered a less invasive diagnostic method in select cases [21].

Management of nasal tophi typically combines surgical excision—serving both for definitive diagnosis and symptomatic relief—with long-term urate-lowering therapy. Adherence to European Alliance of Associations for Rheumatology (EULAR) guidelines is crucial to maintain serum urate levels below 300 μmol/l [22, 23]. In cases of refractory gout, novel therapeutic agents such as AR882 or pegloticase may be considered, although their efficacy and safety specifically for nasal tophi require further investigation [24, 25]. The favorable outcome in our case, with complete resolution and no recurrence at 9 months, underscores the efficacy of this combined approach. Recent studies [10] further demonstrate that treat-to-target urate-lowering strategies can lead to complete tophus resolution as monitored by DECT, emphasizing the importance of consistent long-term management[26].

This report is limited by its single-case design. The proposed diagnostic algorithm and the pathophysiological hypothesis regarding tophus formation in Kiesselbach’s area warrant further validation in larger, multicenter studies. Despite this limitation, our case highlights the critical need for a high index of suspicion for gouty tophi in atypical locations, particularly in patients with a history of gout, even if the presentation is painless.

## Conclusion

Gouty tophi in Kiesselbach’s area of the nasal septum are exceedingly rare. Dual-energy CT is a valuable diagnostic tool, and the proposed “3 + 2” algorithm may facilitate early recognition in clinical practice. The combination of surgical excision and consistent urate-lowering therapy yielded excellent outcomes in this particular case. Further studies are needed to validate the algorithm and to clarify the underlying pathophysiological mechanisms contributing to tophus formation in this unusual anatomical site.

## Data Availability

All data relevant to this case report are included in the article and its supplementary files.
